# From Brazil to the world: a journal with purpose

**DOI:** 10.1590/2175-8239-JBN-2025-E015en

**Published:** 2025-07-18

**Authors:** José A. Moura-Neto, Thyago Proença de Moraes, Miguel Carlos Riella

**Affiliations:** 1 Sociedade Brasileira de Nefrologia, São Paulo, SP, Brazil.; 2 Escola Bahiana de Medicina e Saúde Pública, Salvador, BA, Brazil.; 3 Pontifícia Universidade Católica do Paraná, Curitiba, PR, Brazil.; 4 Fundação Pró-Renal, Curitiba, PR, Brazil.

As we advance through a new biennium and celebrate 65 years since the founding of the Brazilian Nephrology Society, we take this moment to reflect and reaffirm the commitment of the Brazilian Journal of Nephrology (BJN) to the global nephrology community.

**Figure 1:**
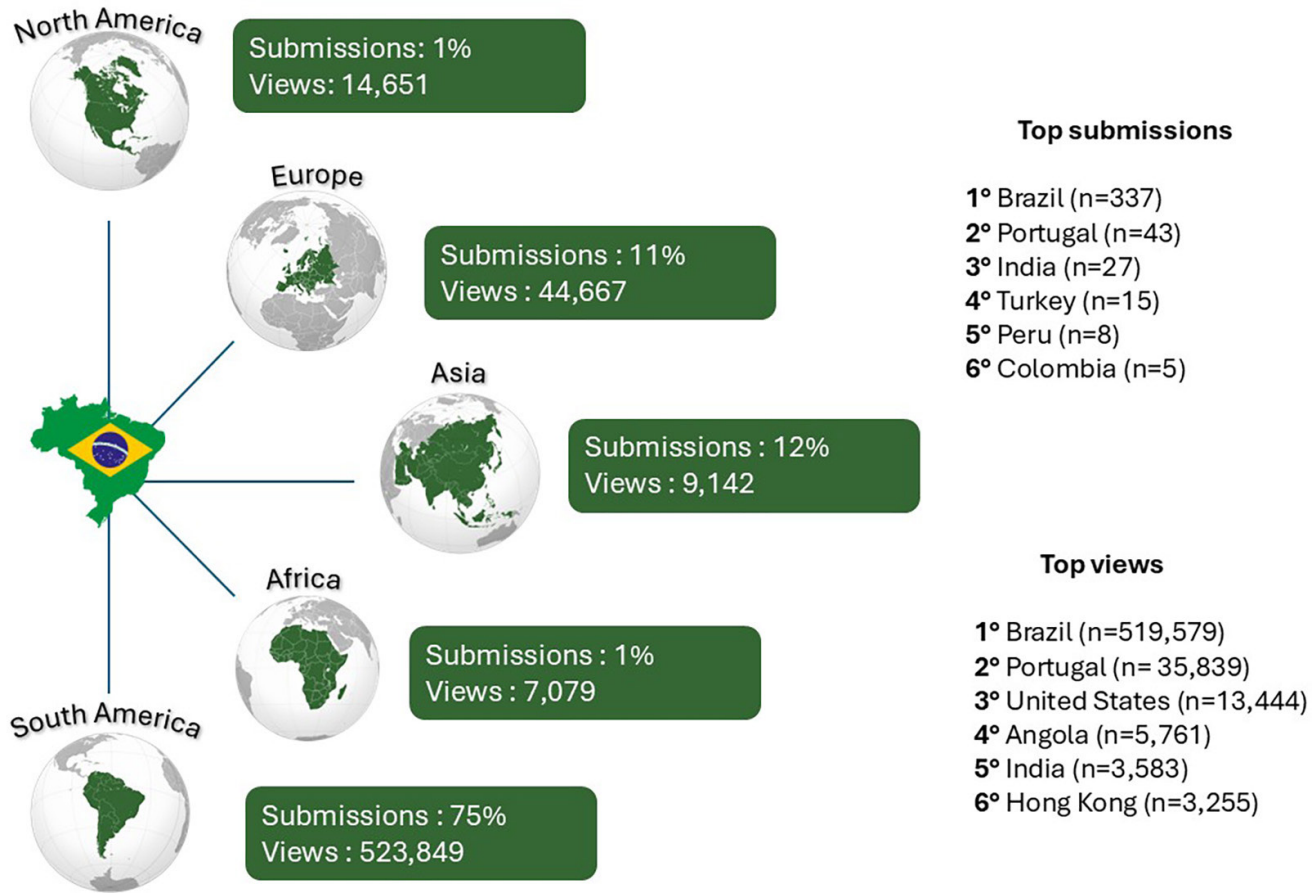
Submissions and views of the *Brazilian Journal of Nephrology* by continent from January 1, 2023, to December 31, 2024.

BJN continued its growth trajectory in the period 2023-2024. We received 472 submissions from 23 countries and published 175 articles across eight regular issues over the course of these two years. In addition, three supplements featuring conference abstracts were released. Our publications reached readers in more than 10 countries, and submissions came from every continent — an extraordinary milestone for a journal rooted in Latin America and financially supported by a national medical society committed to equity, science, and public health. Approximately 75% of the submissions were from South America, with Brazil — unsurprisingly — leading the way. Among the international contributors, Portugal, India, and Turkey were the most prominent sources of manuscript submissions (Figure 1).

BJN is indexed in Scopus, Medline, PUBMED Central, PUBMED, SciELO, DOAJ, and Emerging Sources Citation Index (Web of Science Core Collection™) and is increasingly recognized as a credible, plural, and accessible platform for kidney health research and education in. Founded in 1979, BJN received its first impact factor (1.2) in 2023, rising to 1.3 in 2024^1^.

But metrics — as important as they may be — are not our ultimate goal. Impact must be grounded in integrity. Relevance must be paired with responsibility and visibility must be guided by values. That is why we focus not only on numbers, but also on meaning. Since 2019, BJN has pioneered the regular use of Visual Abstracts in Latin America, accompanying every original article with a concise, easily accessible summary that bridges the gap between science and communication^2^. The journal remains fully open access, with no publication fees for authors or readers, underscoring our belief that knowledge should circulate freely. In a world facing overlapping crises — climate change, political instability, health inequities, and the rising burden of chronic diseases — nephrology must be part of the solution. BJN aims to be a space where diverse voices converge to advance this mission. A journal not just of science, but of purpose – and this editorial is a statement of that purpose.

In 2025–2026, we will continue to welcome submissions that are methodologically sound, socially engaged, and clinically relevant —from large clinical trials to real-world data, from narrative reviews to original articles, from Brazil to the world. Inspired by the Brazilian spirit — resilient, creative, and committed to the community — the aim is for the BJN to remain a bridge between knowledge and action, regions and realities, science and solidarity.

The title of this editorial echoes a slogan born in late 2023 — a silent manifesto of pride that starts on the inside and radiates outward. We invite our readers, authors, reviewers, and editorial collaborators to join us to build this journal together. Our commitment is to uphold BJN as a space of excellence, openness, and meaningful dialogue.

Let us move forward together through this new biennium — step by step, idea by idea, paper by paper.
