# Supplementary material for: From Brazil to the world: a journal with purpose
Source: J Bras Nefrol. 2025 Jul 18;47(3):e2025E015. doi: 10.1590/2175-8239-JBN-2025-E015en (PMC12279283; doi:10.1590/2175-8239-JBN-2025-E015en)
Supplement: Translation

# Translation: Do Brasil para o mundo: uma revista com propósito

**DOI:** 10.1590/2175-8239-JBN-2025-E015pt

**Authors:** José A. Moura-Neto, Thyago Proença de Moraes, Miguel Carlos Riella

**Affiliations:** 1 Sociedade Brasileira de Nefrologia, São Paulo, SP, Brasil.; 2 Escola Bahiana de Medicina e Saúde Pública, Salvador, BA, Brasil.; 3 Pontifícia Universidade Católica do Paraná, Curitiba, PR, Brasil.; 4 Fundação Pró-Renal, Curitiba, PR, Brasil.

À medida que avançamos no novo biênio e comemoramos os 65 anos de fundação da Sociedade Brasileira de Nefrologia, gostaríamos de aproveitar este momento para refletir e reafirmar o compromisso do Brazilian Journal of Nephrology (BJN) com a comunidade nefrológica mundial.

O BJN manteve sua trajetória de crescimento no período de 2023 a 2024. Recebemos 472 submissões de 23 países e publicamos 175 artigos distribuídos em oito edições regulares ao longo desses dois anos. Além disso, foram lançados três suplementos contendo resumos de conferências. Nossas publicações alcançaram leitores em mais de 10 países e as submissões vieram de todos os continentes - um marco extraordinário para uma revista enraizada na América Latina e apoiada financeiramente por uma sociedade médica nacional comprometida com a equidade, a ciência e a saúde pública. Aproximadamente 75% das submissões foram provenientes da América do Sul, com o Brasil - sem surpresa - ocupando o primeiro lugar. Entre os colaboradores internacionais, Portugal, Índia e Turquia foram as principais fontes de submissão de manuscritos (Figura 1).

**Figura 1:** Submissões e acessos ao Brazilian Journal of Nephrology, por continente, no período de 1º de janeiro de 2023 a 31 de dezembro de 2024.

O BJN é indexado no Scopus, Medline, PUBMED Central, PUBMED, SciELO, DOAJ e *Emerging Sources Citation Index* (*Web of Science Core Collection*™) sendo cada vez mais reconhecido como uma plataforma confiável, plural e acessível para pesquisa e educação em saúde renal. Fundado em 1979, o BJN recebeu seu primeiro fator de impacto (1,2) em 2023, aumentando para 1,3 em 2024^1^.

No entanto, as métricas - por mais importantes que sejam - não constituem nosso objetivo final. O impacto deve estar fundamentado na integridade. A relevância deve estar associada à responsabilidade e a visibilidade deve ser orientada por valores. É por esse motivo que nos concentramos não apenas em números, mas também no significado. Desde 2019, o BJN tem sido pioneiro na América Latina no uso regular de Resumos Visuais, acompanhando cada artigo original com um resumo conciso e de fácil acesso que estabelece uma ponte entre ciência e comunicação^2^. A revista permanece com acesso totalmente aberto, sem taxas de publicação para autores ou leitores, ressaltando nossa convicção de que o conhecimento deve circular livremente. Em um mundo que enfrenta crises sobrepostas - mudanças climáticas, instabilidade política, desigualdades na saúde e o aumento do ônus das doenças crônicas - a Nefrologia deve ser parte da solução. O BJN tem como objetivo ser um espaço onde vozes diversas convergem para o avanço dessa missão. Um jornal não apenas de ciência, mas de propósito - e este editorial é uma declaração desse propósito.

Em 2025-2026, continuaremos a receber submissões que sejam metodologicamente robustas, socialmente engajadas e clinicamente relevantes - desde grandes ensaios clínicos até dados de mundo real, de revisões narrativas a artigos originais, do Brasil para o mundo. Inspirado pelo espírito brasileiro - resiliente, criativo e comprometido com a comunidade - o objetivo é que o BJN se mantenha como uma ponte entre conhecimento e ação, regiões e realidades, ciência e solidariedade.

O título deste editorial ecoa um lema nascido no final de 2023 - um manifesto silencioso de orgulho que se inicia internamente e irradia para fora. Convidamos nossos leitores, autores, revisores e colaboradores editoriais a se unirem a nós para construirmos juntos esta revista. Nosso compromisso é manter o BJN como um espaço de excelência, abertura e diálogo significativo.

Sigamos juntos neste novo biênio - passo a passo, ideia por ideia, artigo por artigo.
